# The Analysis of Mammalian Hearing Systems Supports the Hypothesis That Criticality Favors Neuronal Information Representation but Not Computation

**DOI:** 10.3390/e24040540

**Published:** 2022-04-12

**Authors:** Ruedi Stoop, Florian Gomez

**Affiliations:** Institute of Neuroinformatics, University and ETH Zürich, Irchel Campus, Winterthurerstr. 190, 8057 Zürich, Switzerland; fgomez@ini.phys.ethz.ch

**Keywords:** criticality, computation, hearing, listening

## Abstract

In the neighborhood of critical states, distinct materials exhibit the same physical behavior, expressed by common simple laws among measurable observables, hence rendering a more detailed analysis of the individual systems obsolete. It is a widespread view that critical states are fundamental to neuroscience and directly favor computation. We argue here that from an evolutionary point of view, critical points seem indeed to be a natural phenomenon. Using mammalian hearing as our example, we show, however, explicitly that criticality does not describe the proper computational process and thus is only indirectly related to the computation in neural systems.

## 1. Introduction

Collective dynamics of neurons is at the base of the cognitive functions and behavior of higher animals [1,2,3,4,5]. Salient expressions are continued spontaneous activities that modulate neural responses to external stimuli and are often thought to reflect expectations of future stimuli [2]. An observation that has received widespread interest is spontaneous spatiotemporal patterns of neural activity, commonly termed ‘neural avalanches’, that have no characteristic scale, i.e., the distributions of avalanche size and lifetime follow power laws. This scale-free property has approximately been identified in diverse experimental settings, e.g., in electrophysiological in vitro recordings [6,7,8], in in vivo recordings [9,10], in electroencephalogram recordings [11,12] and in functional magnetic resonance imaging [13]. If many—generally relatively simple—interacting elements generate system properties that exhibit power law distributions, this can be taken as a fingerprint of a neighboring critical (transition) point of the collective behavior [14]. Consequently, it has been conjectured that cortical neural systems might operate in the close vicinity of a critical point [15,16,17,18,19] and it has been suggested that, using such a state, the brain might optimize functional properties relevant for information processing (such as information transmission, information capacity and response flexibility and computation) [20,21,22,23]. While the role of criticality in the brain is still actively debated [4,23,24,25,26], we collect and interpret here recent insights from the mammalian hearing system [23,27,28], a fundamental prototype of neuronal function in biological systems.

## 2. Universality and Criticality in Physics

In physics, critical points are related to phase transitions. While phase transitions are often easily captured, a precise definition of ‘phase’ is difficult. Whereas, near the phase boundaries, we may clearly distinguish phases, the ‘territory’ occupied by a phase may not always be as well defined as it is for gases and liquids. Therefore, we say that states (near the phase transition point) that cannot be changed into each other without a phase transition (a thermodynamic singularity) belong to distinct phases. The mathematical description of a statistical system is normally via a partition function $Z=\sum e^{-\beta H}$, where, in physics, *H* represents a sort of ‘energy’ but, more generally, describes the statistical weight of a configuration in the sum over all configurations that the system can realize. If the system size is finite, the partition sum is a finite sum of positive terms. Each term in this sum is analytic in *T* and *H*, so the sum itself is analytic. As *Z* is strictly positive, also when taking the logarithm for arriving at the free energy or the entropy, a finite system cannot exhibit any singularity and, therefore, no phase transition can occur for such systems. A discontinuous phase transition occurs if the ordered phase loses its stability ‘catastrophically’, i.e., when a slight loss of order favors further loss of order, preventing the emergence of an equilibrium state with reduced stability. In the case of continuous phase transitions, in contrast, an equilibrium state with reduced stability of order can exist. Even though fluctuations become very large near a continuous phase transition point, the ordered phase persists until fluctuation becomes indefinitely large. The specific kind of phase transition is therefore often described by the order of the involved singularity: first-order transition for a singularity in the first derivative, of second order if the singularity is from a higher-order derivative of the statistical description of the system.

The typical computational models of phase transitions in physics are spin systems [29]. Given external parameter temperature *T* and the rescaled temperature distance for the temperature at criticality t=(T−Tc)/Tc, the susceptibility χ diverges as $\chi ∼|t{|}^{-\gamma }$, magnetization as $m∼{\left(-t\right)}^{\beta }$, for t<0 (in the absence of an external magnetic field), and the specific heat as $C_{B}∼|t{|}^{-\alpha }$. Note that the occurrence of critical points and the values of the exponents depend on space dimension *D* in which the process takes place, but that always the critical exponent relation α+2β+γ=2 holds (where α+2β+γ>=2 already holds from fundamental thermodynamics (Rushbrooke inequality [14])). Notwithstanding this element of ordering, a veritable zoo of critical exponent values has been obtained, depending on the specific nature of the objects investigated.

Often, mean-field approaches provide a deeper insight into the nature and properties of phase transitions. The basic idea is that, sufficiently away from phase transition points, the equilibrium average of a function of several spins $f(s_{0},s_{1},\ldots s_{i}\ldots ,s_{n})$ may be computed through separately averaging all the spins. If we may assume that fluctuations are not large, i.e., $\langle {s_{k}}^{i}\rangle ∼\langle {s_{k}}^{k}\rangle$, we arrive at $\langle f\left(s_{0},s_{1},\ldots ,s_{n}\right)\rangle ≃f\left(\langle s_{0}\rangle ,\langle s_{1}\rangle ,\ldots ,\langle s_{n}\rangle \right)$. This implies that mean-field approaches can rarely assert anything definitive about phase transitions if fluctuation effects are not inessential: neither the existence, nor, if a transition happens, its order can be safely predicted, although common expectation is that, for d≥4 (especially d>4), the simplest mean-field results are generally qualitatively correct, for fluids and magnets; see [30].

For simplicity and didactical reasons, we have concentrated above on critical phenomena of simple spin-like systems at equilibrium. The ‘portfolio’ of critical phenomena extends, however, far beyond, by including cases of general nonequilibrium systems [31], systems with glassy states [32], mixtures yielding critical lines [5], bifurcation [33,34], percolation [35] systems, cellular automata [36] and more. While still based on the criticality principle, the distinct phenomena follow their proper rules and have their own critical exponents.

## 3. Application to Biology

The term ‘scaling’ embraces fundamentally simple observations that exhibit scale invariance. Scaling of a function or form f(x) by a constant factor *c* leads to a proportional function f(cx)=cf(x), which is possible for $f\left(x\right)=ax^{k}$, leading to $c=c^{-k}$. Such a simple idea is behind the measurement of objects by means of the concept of length. Many biological processes, in contrast, follow ‘allometric scaling’ (small animals look fundamentally different from large ones, since size (length) force and bone strain scale differently). Thus, if we observe true scaling in nature, this points to fundamental laws or processes that do not change across magnitudes of order. Moreover, if the behavior of a substantial class of objects is dominated by these laws in a ‘universal’ manner, a critical point might be responsible for this, since, at a critical point, simple long-range properties dominate over the local properties that may distinguish individual systems. Since power laws are related to scaling properties contained in the physical world, they are ubiquitous (but not necessarily generic). As a prominent case, in complex networks theory, a general preferential attachment principle leads to power laws in the distribution of mesoscopic network indicators, such as network degree, connectivity weight [37,38,39,40], avalanche size or lifetime [41,42,43]. This principle expresses that, for the formation of the underlying networks, attractive forces valid over decades of spatial extensions are responsible (in physics related to, e.g., mass, charge) [44]. A second, additional, fundamental principle, active at the same time, is that real-world connectivity requires space and that this space is, generally, limited. This leads to power laws terminating in a hump (which, upon the network’s growth, moves towards larger network degrees, until the process is stopped by node depletion, an observation that is rather common in power-law-like distributions observed in nature [44]).

Given this situation, why should one be interested in criticality in biology at all? In biology, we often see that sometimes very small variations in the building plan or configuration of the microscopic system can have a huge effect, but also regions exist in which the effects generated by such variations are minimal (see DNA as an example, or, as a more theoretical example, recall how even iterations of two-dimensional maps such as $f\left(\left\{x,y\right\}\right):=\left\{y-|xb-a{|}^{1/2}Sign\left(x\right),a-x\right\}$ generate patterns of very similar appearance for large parameter areas, until, upon a minimal parameter change, this abruptly ends, and an area of changed dynamics with a changed complexity of prediction [45] is entered). This fundamental feature, commonly described as ‘behavior’, has a strong correspondence with the properties of cellular automata, where a simple variation of a rule may preserve or dramatically change the observed behavior [36].

Critical states [14,46] are typically associated with continuous phase transitions at the border between qualitatively distinct dynamical behavior (e.g., ordered vs. disordered dynamics). Due to the diverging correlation lengths at critical states, systems near criticality show high susceptibility to external stimuli, which has been seen as a favorable property of natural systems [15,16,17,18,19,20]. A particularly important role that criticality may play is that it offers a physics-proven perspective to such observations and phenomena, e.g., at criticality [14], we observe classes sharing ‘universal’ properties, in which the individual properties do not matter. A prominent example is the universal Feigenbaum scenario to chaos, which is valid for all systems the dynamics of which have a quadratic leading to nonlinearity. Chaotic—in contrast to ordered—dynamics emerge at the critical point of a period-doubling renormalization and are characterized by the so-called Feigenbaum constants [33,34]. Physics, moreover, also promises that such results can be extrapolated away from the critical point [47], yielding also insights into the nature of the processes that drive the behavior of the class. In the most condensed form, the universality classes condense into so-called ‘critical exponents’ that describe the power laws from a continuous phase transition ‘at criticality’.

The boundary between stably ordered vs. variable adaptive behavior, at which critical points are expected to emerge, has been suggested as the natural ‘habitat’ of biological evolution [48,49]. On the temporal microscopic scale, scale-free avalanches of neuronal firing events have suggested that these networks might preferably operate at criticality, particularly since theoretical studies of artificial neural networks and of cellular automata have highlighted some potential computational benefits of such a state. Presently, the general opinion states that, at a critical state, the brain might optimize functional properties relevant for information processing (such as information transmission, information capacity and response flexibility [20,22,25]), where the precise functional role of criticality has, however, so far only been pinned down for the peripheral auditory system [23].

### 3.1. A List of Prominent Conjectures

As an explanation for the occurrence of power law distributions, a number of hypotheses have therefore been formulated:

Conjecture 1: (SOC) Nature generally self-organizes towards criticality; typical systems are slowly driven at nonequilibrium with many degrees of freedom and strongly nonlinear dynamics [50,51,52,53].

Conjecture 2: A classical model of self-sustained branching describes the avalanches of events in biology, in size *S* and in lifetime *T* as $p\left(S\right)∼S^{-\tau }$ and $p\left(T\right)∼T^{-a}$, where τ=3/2 and a=2 [22].

Conjecture 3: More specifically, dynamical ‘edge of chaos’ and ‘avalanche’ criticality are different sides of the same phenomenon and occur jointly [1].

Conjecture 4: Biological systems, artificial neural networks and cellular automata operate at critical points because of computational benefits. This interpretation, probably motivated by earlier works [54,55,56], is often referred to in neuroscience. 

There are, however, still only a few system examples or theoretical arguments using evolutionary or on directly accessible time-scales that support Conjecture 1. We will show, using neuronal culture development as our demonstration field, that Conjecture 2, originating from the branching model of the border between exploding and dying activity [22] (suggested to rule criticality in many biological and even in some physical systems such as earthquakes [51,57]), might be a good model in some cases, but will otherwise be too restrictive. Easy-to-construct, close-to-biology neural network examples that are at an avalanche critical point, but clearly have a chaotic dynamical character, demonstrate that Conjecture 3 is generally wrong [4]. We will finally exhibit, using results from the field of mammalian hearing (a very ancient neural-like system), that many statements made in neuroscience in the context of Conjecture 4 tend to be imprecise and to miss the true nature of the phenomenon.

In applications of the concept of criticality to neuroscience and in particular to the brain, it has been observed that the obtained experimental scaling exponents fail to fulfil the expectation of a single ‘natural’ universality class (similarly to the physics case). In early experimental studies [6], the scaling exponents associated with avalanche size and lifetime distributions, $p\left(S\right)\;∼\;S^{-\tau }$ and $p\left(T\right)\;∼\;T^{-\alpha }$, respectively, appeared to be τ≈1.5 and α≈2.0. These values match the theoretically expected critical exponents of the mean-field critical branching process [58,59], suggesting that biological networks operate at a critical point characterized by a marginal propagation of activity, separating two phases of quickly decaying and exploding runaway activity [6]. Following studies, however, soon revealed scaling exponents of a considerable variation (e.g., τ∈(1.5,2.6) [6,16,60,61]). Largely, the origins of the deviations were seen as artefacts, induced by deficiencies of either the experimental measurements (e.g., spatial undersampling of neural activity [19,62]) or of the computational approaches involved in the avalanche extraction procedures [6,19]. Results from a recent study of dissociated hippocampal neurons co-cultured with glia cells (prepared from newborn P0 Sprague Dawley rats) that, by using optical imaging, largely eliminated the problem of spatial subsampling, indicated, however, the existence of two distinct criticality regimes, with different sets of critical exponents [63]. While one of the critical regimes indeed matched the signatures of the aforementioned critical branching process, the other revealed the presence of substantially larger critical exponents (τ≈2.2, α≈3.3). Probably following the credo of a single critical point, the observation of the second critical regime was interpreted as an artefact of a specific pharmacological manipulation (by 5M4Hfolate), which left open whether importance should be attributed to the second critical regime at all. A more recent study with neural cultures on a multi-electrode (‘MEA’) chip [5] showed, however, in full rigor (satisfaction of the crackling noise relationship [64,65] and scaling function collapse), that, in addition to a more standard critical state of exponents τ≈1.65, a≈2.0, a critical regime with power law exponents τ≈2.2, a≈2.8 exists, at an earlier stage of the development. Corresponding modeling verified these states and suggested, moreover, the existence of a critical line along which the culture’s states meander during their development [5].

It has similarly widely been assumed that criticality in biology would be strong enough to unify ‘avalanche’ and ‘dynamical edge of chaos’ criticality [1]. However, a recent study [4] demonstrated that it is quite possible to observe avalanche criticality without any evidence of edge of chaos criticality, under biologically reasonable model assumptions. In that study, based on a recurrent neural network of more realistic neurons compared to what had been used previously, it was shown that the largest Lyapunov exponent continued to be positive as the network was tuned from subcritical to critical and to supercritical avalanche behavior.

### 3.2. Cochlear Prototype of Neural Circuits

The sensory elements of the animal nervous system work in a remarkably uniform way in the following sense. Neurons, as well as the evolutionary older hair-type sensor cells, pick up a specific range of external signals (often connected to frequency properties), amplify the latter in a strongly nonlinear manner and transmit the amplified signal further down the sensory pathway. Stimulations can be of variable type: chemical, electrical (neurons) or physical (hair cells). Their preference for specific signals (embracing generally a certain bandwidth thereof) is encoded in their building plan (e.g., sensor size, architecture, physical neuronal membrane or hair properties, etc.); their readiness towards picking up signals is commonly described by the term ‘excitability’. This term measures, in a sense, how far the sensor’s state is from a state where the sensor’s amplification process would generate, even in the absence of an external stimulation, a (in most cases, undesired) signal. In addition to depending on internal factors, excitability can generally be influenced from outside (e.g., as a widely applied experimental technique, by chemicals).

‘Computation’ is often used in the biological context in a sloppy manner, but can rigorously be defined as an information destruction process. A paradigm for this view is provided by the logical ‘OR gate’, where the inputs are 0/1 pairs and the output is a single 0/1 signal. If we obtain 1 as the result, we no longer know where the result came from, i.e., previously available information was destroyed. By stripping information from a potential connotation of ‘usefulness’, a general notion of computation can therefore be made precise by measuring the amount of information destroyed in this process [45,66]. For the moment, it suffices to retain that, in this view, sensory elements already perform computation, by the selection of specific information from a range of available information. In the following, we will consider under what conditions systems of sensory elements maximize computation.

**Figure 1:**
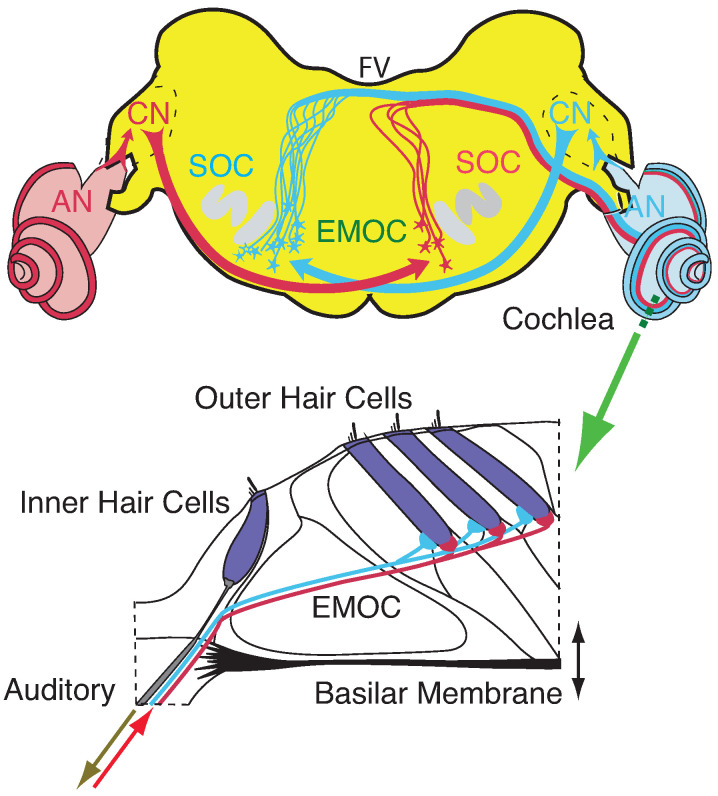
Cochlea’s embedding into mammalian neural circuitry [78,79]. Upper panel: Brainstem section (CN: cochlear nucleus, EMOC: medial olivocochlear efferents, SOC: superior olivary complex, FV: fourth ventricle). Lower panel: Section (dashed green line) of organ of Corti. EMOC stimulations tune the cochlea’s sensitivity (see section ‘Effects of computation’).

The mammalian hearing system, an evolutionary, very ancient prototype of neural-like systems, will be our simple, yet realistic, paradigm for understanding how criticality is involved in neural and neural-like biological computation. Across the mammals, apart from the distinct frequency ranges covered, the construction of the hearing system is exceptionally uniform [44]. It can be argued that the mammalian cochlea is the result of evolutionary optimization driven by the need of a variable-frequency, wide-range, resource-sparing sensor, and that this may have been one of the elements responsible for the success of this class [44]. The working principle of the mammalian cochlea is as follows: arial sound pressure waves arriving from the outer ear at the tympanial membrane are converted by means of the ossicles into waves on the basilar membrane embedded into a shallow fluid canal. Attached to the basilar membrane are outer hair cells (‘OHC’): frequency-specific amplifiers of wave components corresponding best to their preferred frequency FC. Waves first cross membrane areas where outer hair cells respond to high frequencies in the wave, from which the wave proceeds towards basilar membrane areas with OHCs that amplify lower-frequency wave components. At its ‘preferred’ area on the basilar membrane, the strongly amplified wave component is recorded by the so-called inner hair cells and the result is sent up the auditory pathway. After leaving this area, the amplified wave components are attenuated through viscous friction of the cochlear fluid, and the remainder of the wave propagates further down the cochlear duct. Additionally, the hearing organ is embedded in external circuitry, allowing for efferent nerve stimulations (from the brain down to the sensor, labeled in Figure 1 by ‘EMOCS’). The effect of such inhibitory stimulations is reduced amplification by the targeted OHCs. The implementation of the biologically correct OHC amplification profiles (in frequency and stimulation strength, and in external efferent stimulation dependence) is one central issue in cochlear modeling.

In a biophysically mesoscopic manner [67,68], the mammalian cochlea can be modeled by a directed sequence of amplifier circuits (‘sections’), each one optimized around a particular frequency (center frequency ‘CF’) but located below a Hopf bifurcation. A suitably chosen number of sections guarantees, by means of overlapping frequency bandwidths of sections, a continuous frequency amplification profile. The level of excitability of each amplifier is cast in terms of a one-dimensional section parameter μ describing how far the circuit’s resting state from the Hopf bifurcation point is (above bifurcation, self-generated oscillations would emerge [69]). Models of attached inner hair cells and of the auditory nerve complete the modeling of the peripheral hearing system [70,71,72,73]. We emphasize that, as a consequence of the amplifier’s nonlinearity, the superposition picture (fundamental for the ‘piano picture’ of the cochlea) does not hold.

**Figure 2:**
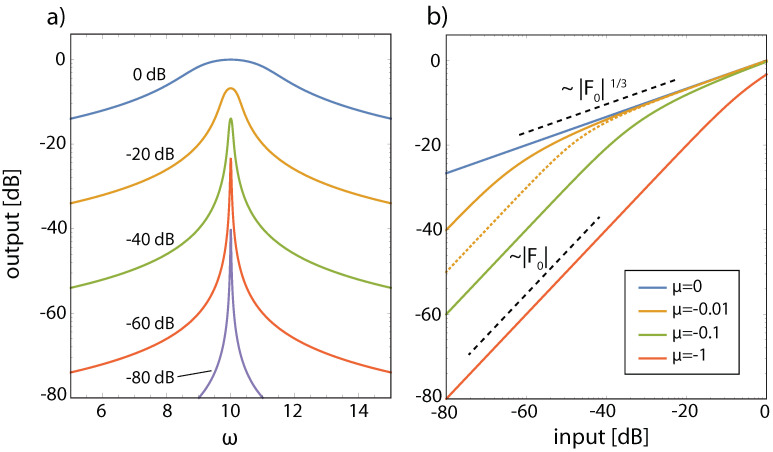
Hopf small signal amplifiers describe the effect of outer hair cells (Hopf frequency $\omega_{ch}=10$, dB output (input) in terms of $10\log_{10}{\left|a\right|}^{2}$ ($10\log_{10}{\left|F_{0}\right|}^{2}$)). (**a**) Output curves as a function of the input frequency ω for different values of $|F_{0}|$ between $10^{-4}$ and 1 and μ=−0.01. (**b**) Output at resonance ($\omega =\omega_{ch}$) for input $F_{0}$ using different μ-values. Dotted: off-resonance curve (ω=9.97, μ=−0.01), for comparison. Note that the amplifier is subcritically tuned, not critically, and isolated from the cochlear environment.

**Figure 3:**
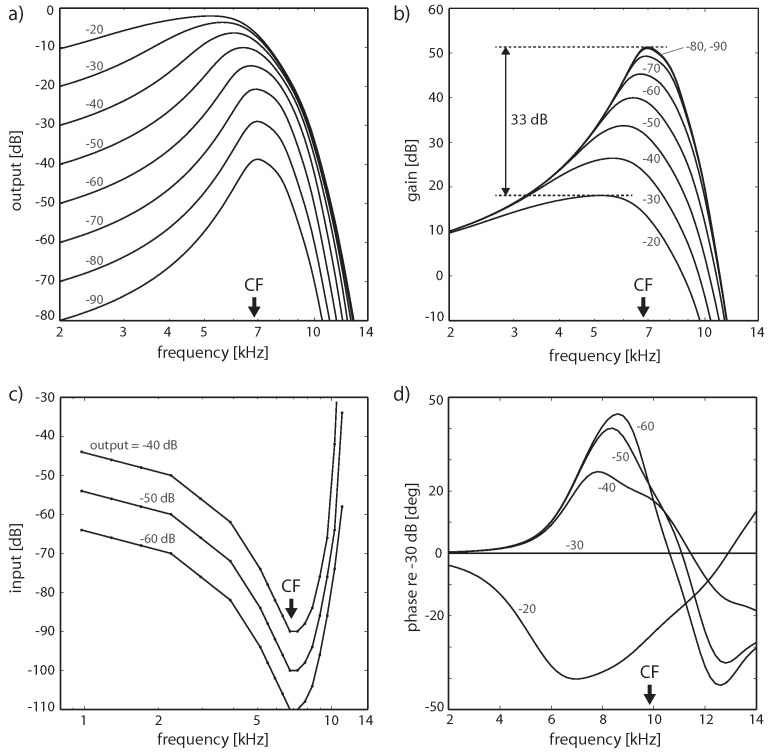
‘Close-to-biology small-signal’ amplifier implementation, including subcritical tuning and influence of cochlear fluid (endolymph). Hopf cochlea response to pure tones. (**a**) Response in dB, (**b**) gain in dB; a difference of 33 dB in peak gain for two input levels differing by 70 dB corresponds to observations in chinchilla (32.5 dB, or slightly higher, between 20 and 90 dB SPL curves [80]). (**c**) Tuning curves for fixed output levels, (**d**) Phase for different input levels relative to −30 dB. (Cochlea discretization covering 14.08–0.44 kHz (20 sections); output at section 5 (CF = 6.79 kHz) for (**a**–**c**) and section 3 (CF = 9.78 kHz) for (**d**). Small numbers denote input levels in dB).

**Figure 4:**
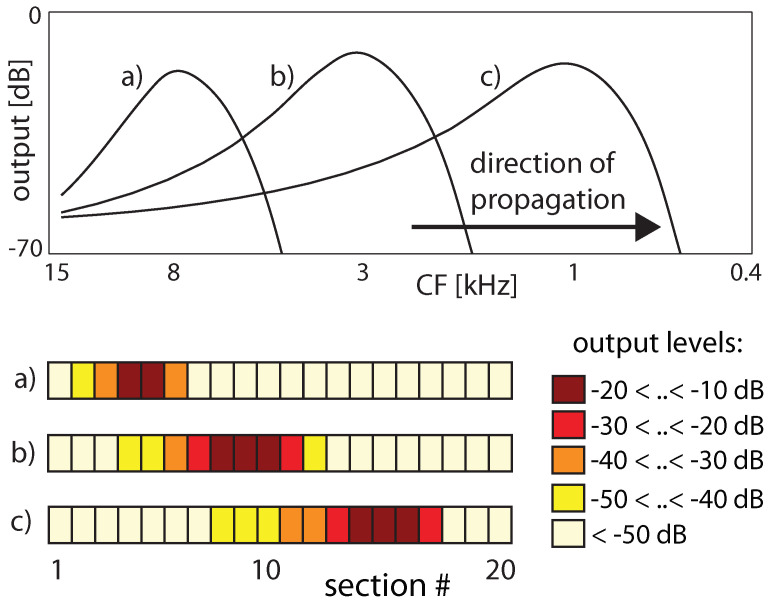
Traveling wave along the cochlea, from the stapes side (hair cells responsive towards high-frequency stimulations) towards the apex (hair cells responding to low frequencies). Input frequencies are 8, 3, 1 kHz in (**a**–**c**), respectively. Upper panel: Extrapolated continuous excitation, lower panel: activation patterns on the artificial cochlea.

**Figure 5:**
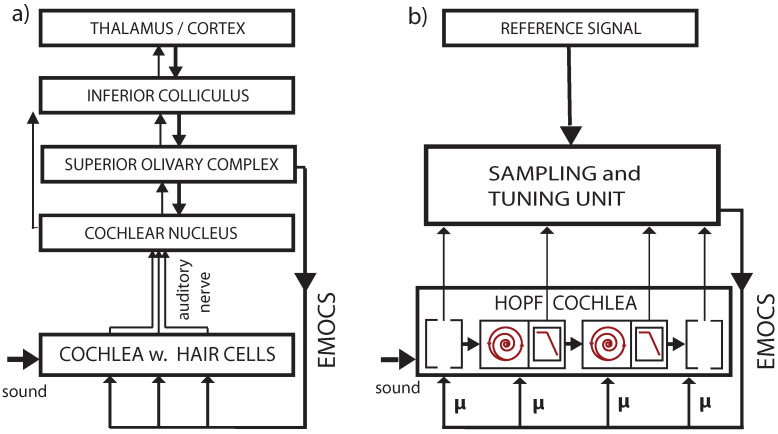
(**a**) Biological vs. (**b**) artificial implementation of the hearing–listening circuit. Listening is a dedicated activity that represents a particular computational effort, involving ‘EMOCS’ (efferent medial olivocochlear stimulations), cf. Ref. [81].

More specifically, the key for understanding hearing is the outer hair cells’ ’small-signal amplifier’ property. First demonstrated by Wiesenfeld et al. [74,75], systems close below a (period-doubling) bifurcation act as ideal small-signal amplifiers: signals of small amplitudes with a specific ‘critical’ (better, ‘center’ frequency CF) are strongly amplified, whereas strong signals or signals with from CF strongly deviating frequencies are not amplified. This phenomenon applies to bifurcations in general, and to systems at a Hopf bifurcation in particular; see Figure 2 and Figure 3. From early publications [76,77], the expectation might have emerged that the mammalian hearing system might be fully described in terms of the small-signal amplifier property. However, only the precise tuning of the Hopf amplifiers along the cochlea together with the properties of the cochlear fluid generate the correct shallow surface waves [28,68]; see Figure 4. Moreover, for the understanding of how mammalian hearing and listening works, the embedding of the hearing sensor into the cortical circuitry (see Figure 1 and its schematic representation in Figure 5) plays an essential role, as we will demonstrate in the sequel.

**Figure 6:**
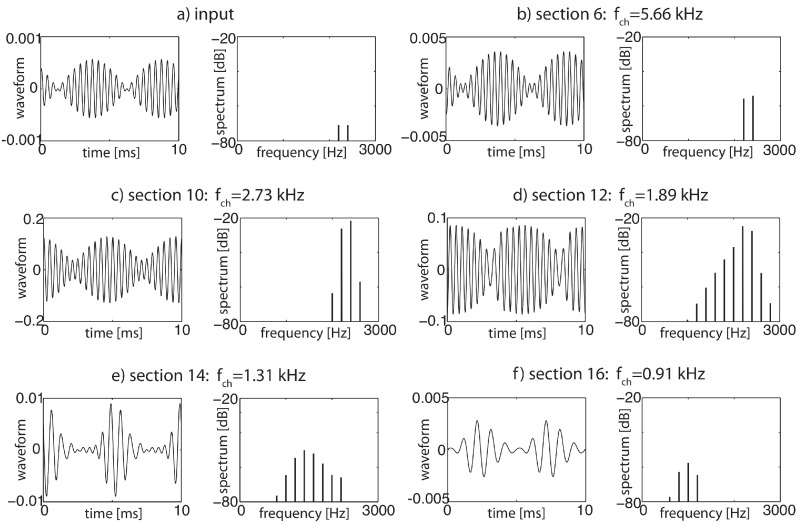
From two pure input tones of $f_{1}=2200$ and $f_{2}=2400$ Hz, each at −74 dB sound level (**a**,**b**), additional ‘combination’ tones are generated (cf. (**c**–**f**)), due to the nonlinearities of the amplifiers at differences of the input frequencies, of essentially exponentially decaying amplitudes.

As has already been mentioned, an important consequence of the strongly nonlinear nature of the amplifiers is that the superposition principle valid for linear amplifiers does not hold. Interaction terms are formed between the amplifiers that can be measured on the basilar membrane and in the simulations; see Figure 6. These combination oscillations—if above the hearing threshold perceived as combination tones (‘CT’)—emerge for two pure tones at frequencies $f2>f_{1}$ at difference frequencies $\left(f_{2}-f_{1}\right)$, $\left(2f_{1}-f_{2}\right)$, $\left(3f_{2}-2f_{1}\right)$, etc., and, less prominently, at sum frequencies $\left(f_{2}+f_{1}\right),\left(2f_{2}+f_{1}\right)$, with approximatively exponentially decaying amplitudes [28]. The general corresponding condition on positive integers is $k^{\prime }+k^{\prime \prime }-k^{\prime \prime \prime }=l$, where *l* corresponds to the specific frequency considered ($\omega_{l}=l\omega_{0}$).

**Figure 7:**
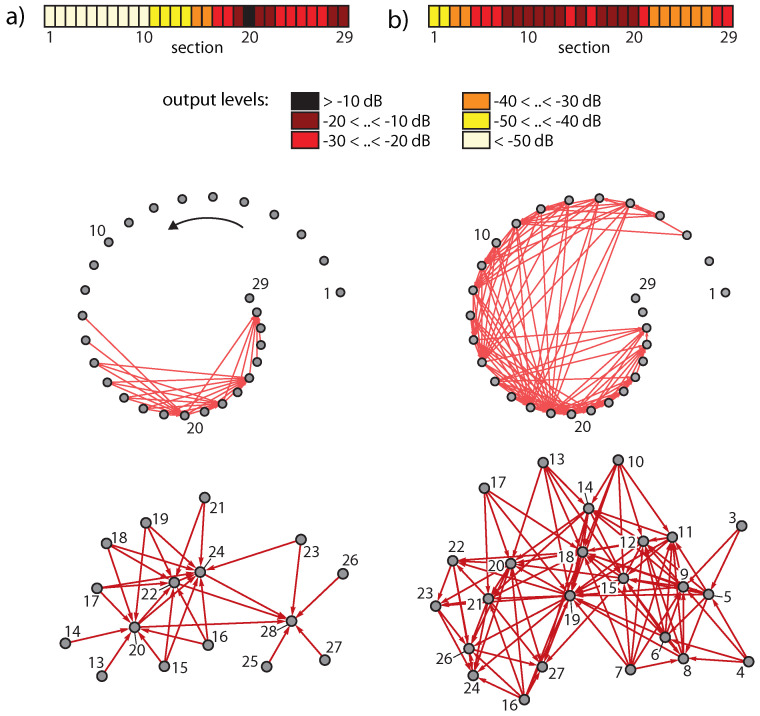
Activation networks from (**a**) two pure tones (3/8, 1/2 kHz), (**b**) two complex tones (2, 3.35 kHz, 5 harmonics each).

The generation of these additional frequencies can be seen as a branching process and can be cast in a network scheme, where sections generating ‘artificial’ tones in the cochlea not contained in the input signal are at the endpoints of arrows; see Figure 7. In this network, nodes below the physiological hearing threshold (‘unactivated’ nodes) have been eliminated [23].

**Figure 8:**
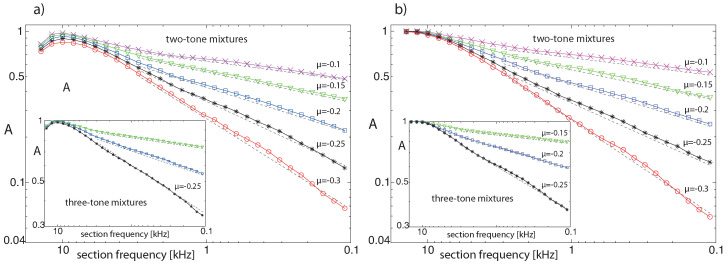
Cochlea activity *A* using uniformly chosen amplifier parameter values μ (range 0.1–0.3) and *N* = 10,000 pairs of random base frequency complex tones, from (**a**) sound levels random from the interval (−80,−40) dB (rms) per tone, (**b**) from −60 dB fixed sound levels (dashed power law guidelines, exponents β from maximum likelihood estimation: (**a**) β=0.6,0.44,0.3,0.2,0.13, (**b**) β=0.64,0.43,0.29,0.2,0.12 (bottom to top lines)). Insets: Three-tone results.

The ‘activity’ at cochlea sections j=1,…,29 can be defined as

(1) $$A\left(j\right)=\frac{1}{N}\sum_{i}^{N}\Theta \left(f_{i},j\right),$$

where $f_{i}$ denotes the input frequency (or frequencies) of stimulation experiment *i*, *N* is the total number of trials, and $\Theta (f_{i},j)$ is 1 if the output at section *j* exceeds the hearing threshold and 0 otherwise. For pure-tone input uniformly sampled from a (non-logarithmic) frequency interval, the average activity *A* follows a power law of exponent one (each section is activated by a proportion of frequencies corresponding to the ‘bandwidth’ of the section). Two- or three-tone inputs, however, lead to additional CT-generated activity (see Figure 8 for the results over all cochlea sections on log-log scale for different μ settings, for fixed or random input strengths). In all cases, we observe power laws $A\propto f^{\beta }$ with exponents 0<β<1, where the results from two-tone inputs essentially coincide with those from three-tone inputs.

### 3.3. Effects of Computation

**Figure 9:**
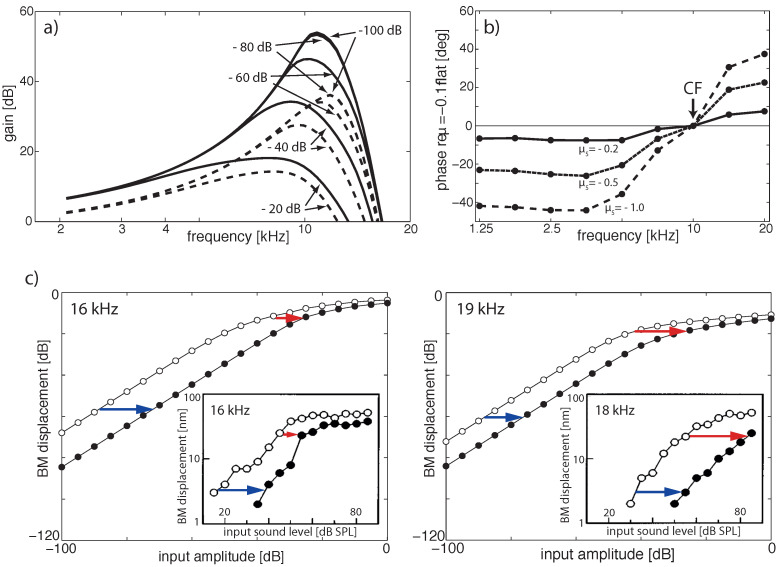
EMOCS effects: (**a**) Gain isointensity curves at section 5 ($f_{ch}=1.42$ kHz) without (solid lines) and with (dashed lines) EMOC input. From flat tuning (μ=−0.1 for all sections), EMOCS is implemented by shifting $\mu_{5}$ to −1.0 (−80 and −100 dB lines collapse). (**b**) Corresponding phase shift at section 5 (phase delays for frequencies below CF, phase leads above CF). (**c**) Comparison to animal data: 16 and 19 kHz pure-tone EMOCS (left and right, respectively) implemented by a shift from a flat tuned cochlea from $\mu_{2}=-0.05$ to $\mu_{2}=-0.5$ lead to BM level shifts at section 2 ($f_{ch}=16.99$ kHz) from open circles to full circles. Insets: Corresponding experimental animal data [82].

**Figure 10:**
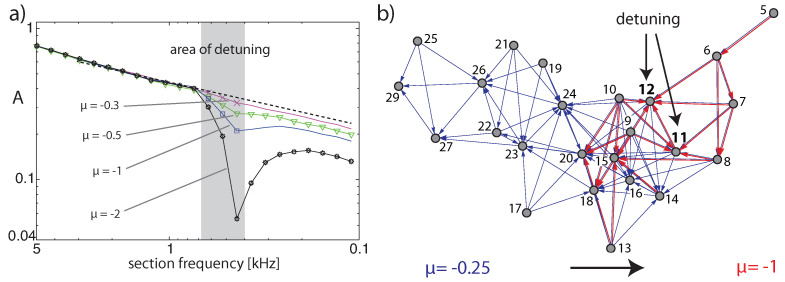
Effects by EMOCS: (**a**) Activation distributions of the experiment of Figure 8 after detuning Hopf sections 19,20,21 from $\mu_{19,20,21}=-0.2$ (dashed), jointly to $\mu_{19,20,21}=-0.3,-0.5,-1,-2$, respectively, at input level −60 dB. (**b**) Detuning of sections 11,12 from $\mu_{11,12}=-0.25$ jointly to $\mu_{11,12}=-1.0$, for the input of two complex tones at −70 dB rms each, with $f_{0}=1331,2120$ Hz and five harmonics.

Understanding biological hearing necessitates the inclusion of the effect by EMOCS [78,79]. We first demonstrate that our modeling reliably reflects the biological effects by EMOCS (see Figure 9) and highlight in Figure 10 that, at the level of the whole sensory organ, EMOCS-changed individual amplifier behavior has striking effects.

**Figure 11:**
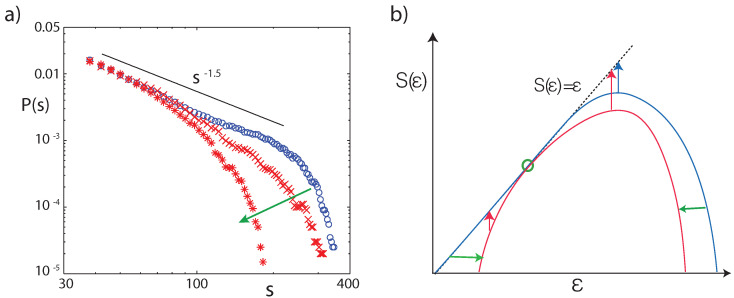
(**a**) Network size distributions (40,000 stimulations with two complex tones, random amplitudes) for flat tuning (blue), and after detuning two frequency bands (sections 15–16, 19–21) from μ=−0.25 to μ=−1.0 (red crosses), and μ=−2.0 (red stars). (**b**) In the thermodynamic formalism, the observability *O* of an invariant measure ε decays with time *t* as $O\left(\varepsilon ,t\right)∼e^{t(\varepsilon -S(\varepsilon ))}$ (red and blue arrows). States represented by entropy values on the diagonal ε=S(ε) do not experience any temporal decay. Blue: Entropy function S(ε) of systems with power law distribution characteristics. Red: Entropy function associated with non-power-law distributions, as the result of focusing on a particular measure (green circle), where horizontal green arrows symbolize the effect by EMOCS (adapted from Ref. [23]).

At −60 dB input level (the typical strength of human speech) and under the absence of EMOCS, the size *s* distribution of the number of network links triggered by two complex tones of random amplitude and frequency follows the typical critical branching network paradigm with exponent a=3/2; see Figure 11a. Stronger input (−50 dB) would yield distributions that are typical for supercritical states. Under the influence of EMOCS, the previous power law changes into a subcritical distribution. These findings indicate that the predisposition of the hearing system towards receiving unbiased information at the most relevant working condition is at criticality, whereas dedicated listening implemented by EMOCS will naturally lead to a subcritical distribution.

In the following, we will show that the predisposition towards the ‘preferred working conditions’ does not naturally imply ‘optimal computation’. Computation is related to the partial destruction of information of a certain degree of complexity. Earlier [45], we posited that complexity is in its nature a complexity of prediction, i.e., is related to a difficulty of the prediction of a future value based on past observations. The ‘richer’ the information produced by the process, the more difficult the prediction process is: it is the appearance of the unexpected that is the hallmark of complexity. For measuring the complexity of a dynamical system, the appropriately scaled integral of the entropy function should therefore be used; the application of this measure to distinct classes of dynamical behavior has revealed its great potential [45]. In particular, regular processes have zero complexity, as well as entirely random ones. Highest complexity is therefore obtained from dynamics based on a continuum of ‘observable measures’, expressed by the power law distribution (the blue curves in Figure 11). How is now computation related to this complexity? By computation, the complexity of prediction is reduced, where the amount of this reduction provides a measure of the performed computation. In the present case of hearing, the ‘natural power law ground state’ without EMOCS indicates the absence of computation, whereas the states under EMOCS (Figure 10 and Figure 11) express that computation has taken place.

### 3.4. Real-World Example of EMOCS-Guided Computation

**Figure 12:**
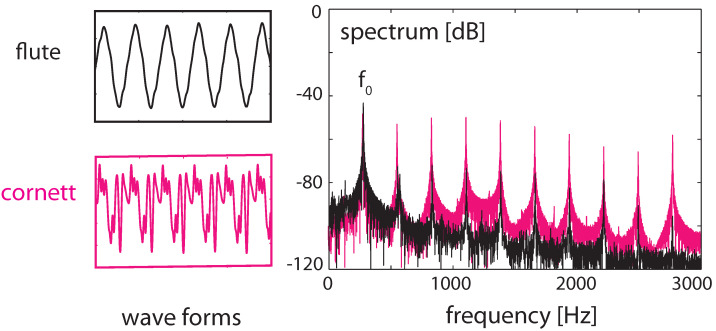
Sounds of a cornett and a flute at the same fundamental frequency $f_{0}$ (**left**), superimposed (**right**), static case.

In the final section, we corroborate that EMOCS processes are naturally associated with real-world computation, by showing the crucial role that EMOCS can play in the separation (or the ‘identification’) of sounds from mixtures of sounds [27]; see Figure 12.

To achieve the separation, the listening process recalls a previously acquired subset of excited amplifiers characteristic for the signal to extract, and disposes the parts of the spectrum that are not associated with the desired signal. This process is implemented by means of EMOCS: nerves leading from the brain to the cochlea via medial olivocochlear stimulation suppress the efficacy of sections unrelated to desired signals by, technically speaking, pushing corresponding Hopf amplifiers further away from the point of bifurcation (for the correctness of this translation from biology to the model, recall Figure 9).

**Figure 13:**
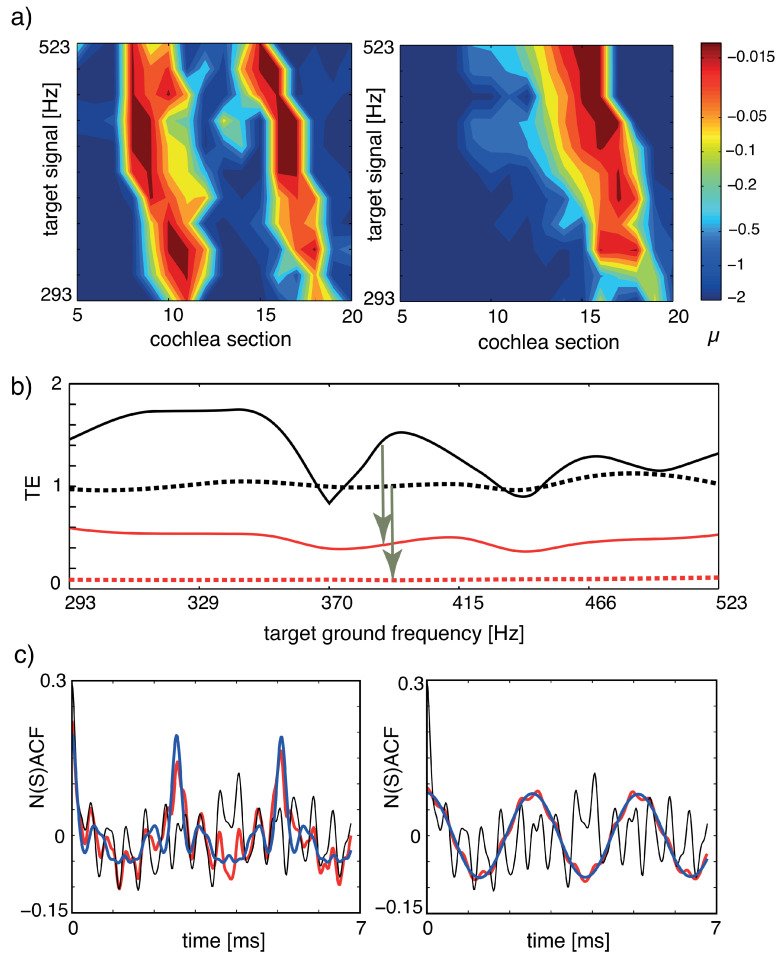
Separation of sounds, dynamic case, where the target instrument changes the height of the generated tone: (**a**) Tuning patterns, dynamical case. Colors indicate the Hopf parameter values of the sections. Left: Cornett vs. flute (disturber). Right: Flute vs. cornett (disturber). (**b**) TE for the two target signals of (**a**). Black: flat tuning. Red: μ-tuning. Full: cornett target, dashed: flute target. (**c**) NSACF, NACF for the two target signals of (**a**,**b**) at a chosen target ground frequency. Black: flat tuning. Red: μ-tuning. Blue: target signal. Targets at 392 Hz, disturbers at 2216 Hz. From Ref. [27].

In Figure 13, we report the result of our implementation of the listening process, where panel (a) shows how the tuning of the amplifiers changes, as the target object (the musical organ) increases its fundamental frequency in time. To assess how close we arrive to the target, we evaluate our tuning error measure TE that has the expression

$$TE\left(x,y\right)=\frac{||norm.\left(\sum_{i}ACF\left(f_{i}\left(x+y\right)\right)\right)-NACF\left(x\right)|{|}_{2}}{||norm.\left(\sum_{i}ACF\left(f_{i}\left(x+y\right)\right)\right)-NACF\left(y\right)|{|}_{2}},$$

where $f_{i}$ denotes the output at section *i* of the cochlea and the summations extend over the *N* sections. NACF is the full normalized summary autocorrelation function accounting for all sound characteristics (such as, e.g., timbre); to measure how strongly a mixture of two input sounds *x*, *y*, is biased towards component *x*, we use the Euclidean distance between the mixture’s NACF (‘NSACF’) and the target signal *x*’s NACF, divided by the Euclidean distance between the mixture’s NSACF and the undesired signal *y*’s NACF. TE values are between 0 and *∞*, where TE=0 indicates a perfect focus and a larger TE a less successful target focus. If one source dominates the mixture, then TE values below unity may emerge even before tuning. Panel (b) exhibits the implemented processes’ efficacy also for time-varying target signals. For fixed target ground signals, panel (c) shows how close we come in terms of NACF (red) to the target signals (blue).

**Figure 14:**
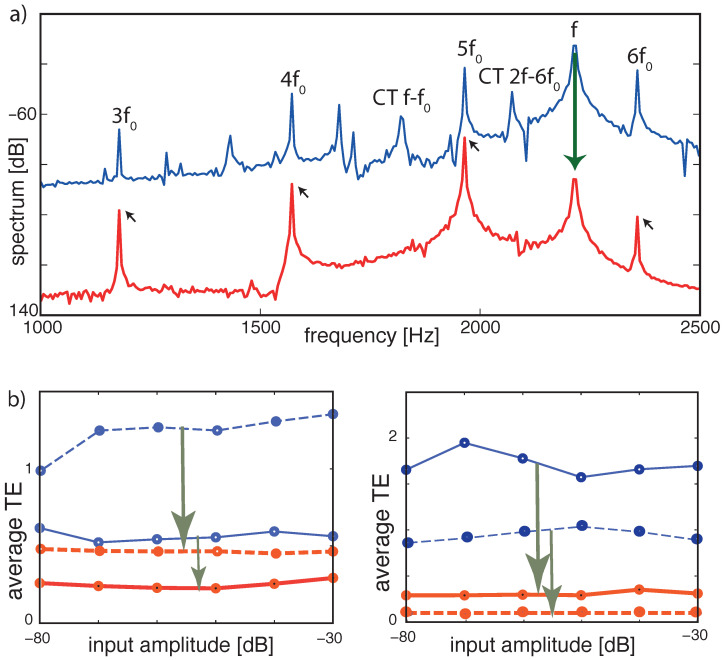
TE improvement by μ-tuning, static case. (**a**) Frequency spectrum at section 8 (CF=1964 Hz). Blue: Flat tuning (−80 dB, target cornett $f_{0}=392$ Hz, disturber flute f=2216 Hz). Cross-combination tones (CT, two explicitly labeled) between the flute’s fundamental *f* and higher harmonics of the cornett are clearly visible. Red: Optimized tuning. *f* (flute) and cross-combination frequencies are suppressed, leaving a harmonic series of the target (small arrows). (**b**) Averaged TE over 13 different fundamental target frequencies (steps of 1 semitone) demonstrates input amplitude independence. Blue lines: flat tuning. Red lines: optimized μ-tuning. Left panel: (full lines) target sound cornett (277 to 554 Hz), disturbing sound flute (at 277 Hz); (dashed lines) same target but flute at 2216 Hz. Right panel: same experiment with target and disturber interchanged. TE improvements: arrows in (**b**). From Ref. [27].

More information can be extracted from the static case at the level of the signals’ spectra, evaluated at variable signal strengths, demonstrating how the combination tones among the two signals are suppressed; see Figure 14a. Moreover, upon variation of the input amplitudes of the two signals, the tuning errors remain small; see Figure 14b.

### 3.5. Conclusions

We have taken mammalian hearing, an evolutionary ancestor of neural systems, as our example to clarify the relation between criticality and computation. Sounds arriving at the mammalian hearing sensor generate, as a consequence of the amplifier’s nonlinearity, networks of activations, quite similar to how stimulations of the nervous system propagate through the neural networks following stimulation-specific pathways. We showed that critical states of the hearing system (expressed by power law activation distributions on the network) correspond to unbiased information uptake (cf. Figure 8). In audition, this state roughly represents what we describe as ‘hearing’. The process of ‘listening’, in contrast, corresponds to a computational state, expressed by the ‘destruction’ of parts of the available information complexity (cf. Figure 10). In this state, the corresponding distributions no longer have the power law form required by criticality (cf. Figure 11). In the final section, we gave an explicit demonstration that the computational process guided by EMOCS enables mammals to identify auditory objects within a cocktail-party environment. The result of the computation is reduced information complexity that the nervous system then uses in further processing steps.

This interpretation has a psychoacoustic correspondence. Asking what combinations of two input sounds are most appreciated by humans (and, likely, more generally by mammals), we found that tone combinations that generate the smallest (weighted) activation networks are judged to be the most pleasant ones. This finding supports that the human mind prefers simple information (as provided by EMOCS-tuned activation networks) over complex information (exemplified by untuned critical activation networks). Indeed, from the evolutionary perspective, it is plausible that living systems should prefer simple signals: signals that are easily perceived, processed and interpreted.
